# Photoconductivity and photoluminescence under bias in GaInNAs/GaAs MQW p-i-n structures

**DOI:** 10.1186/1556-276X-7-539

**Published:** 2012-09-28

**Authors:** Hagir M Khalil, Ben Royall, Simone Mazzucato, Naci Balkan

**Affiliations:** 1 School of Computer Science and Electronic Engineering, University of Essex, Colchester, CO4 3SQ, UK

**Keywords:** p-i-n diodes, GaInNAs/GaAs, Multiple quantum well, Dilute nitrides

## Abstract

The low temperature photoluminescence under bias (PLb) and the photoconductivity (PC) of a p-i-n GaInNAs/GaAs multiple quantum well sample have been investigated. Under optical excitation with photons of energy greater than the GaAs bandgap, PC and PLb results show a number of step-like increases when the sample is reverse biased. The nature of these steps, which depends upon the temperature, exciting wavelength and intensity and the number of quantum wells (QWs) in the device, is explained in terms of thermionic emission and negative charge accumulation due to the low confinement of holes in GaInNAs QWs. At high temperature, thermal escape from the wells becomes much more dominant and the steps smear out.

## Background

Dilute nitride research has sparked considerable interest from fundamental physics to industrial applications, and nowadays, several devices based on GaInNAs/GaAs heterostructures are commercially available [1-7]. The interest on this material started from the discovery that adding small amounts of nitrogen to GaAs and GaInAs resulted in a relatively large redshift in bandgap [8], leading to the realisation of 1.3- and 1.55 μm wavelength devices [9] with strong electron confinement with the use of the well-established GaAs technology.

Extensive work has been carried out on dilute nitrides, and the demonstration of dilute nitride-based LEDs, lasers [10-12] and solar cell devices [13] has already been achieved. In a recently published study [14], we observed several oscillations in the current-voltage (*IV*) characteristics of p-i-n GaInNAs/GaAs multiple quantum well (MQW) structures at low temperature under illumination. By performing the experiment at different photon wavelengths, it was established that the optical transitions in GaInNAs quantum wells were the origin of these oscillations. In this paper, we further investigate the oscillations by studying at the photoluminescence under bias. These results give a more complete understanding of the underlying mechanisms such as thermal escape, trapping, recombination and charge accumulation.

## Methods

The structure studied was a Ga_0.952_In_0.048_N_0.016_As_0.984_/GaAs p-i-n photodiode grown by molecular beam epitaxy (MBE) on an n-doped (100) oriented GaAs substrate. The intrinsic region consists of 10 undoped GaInNAs QWs with varying thickness from 3.8 to 11 nm. The wells were separated from each other by 20 nm thick and from the bulk region by 40 nm intrinsic GaAs barriers. The active region is sandwiched between a 250 nm Be p-doped GaAs layer with doping density of 2 × 10^18^ cm^−3^ and a 600 nm Si n-GaAs layer with 5 × 10^17^ cm^−3^ doping density. The sample [see Additional file 1 was fabricated in the shape of a mesa-structure, with top circular aperture of 1 mm diameter. Further details about growth and fabrication can be found in our previous publication [15].

## Results and discussion

**Figure 1:**
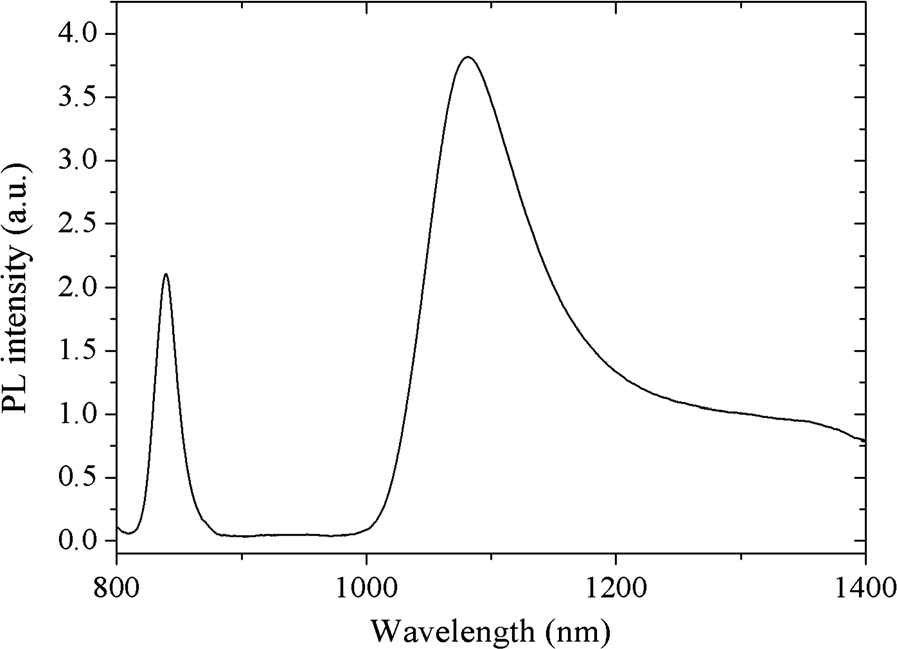
**PL emission from the investigated sample at *T*** = **100 K.**

**Figure 2:**
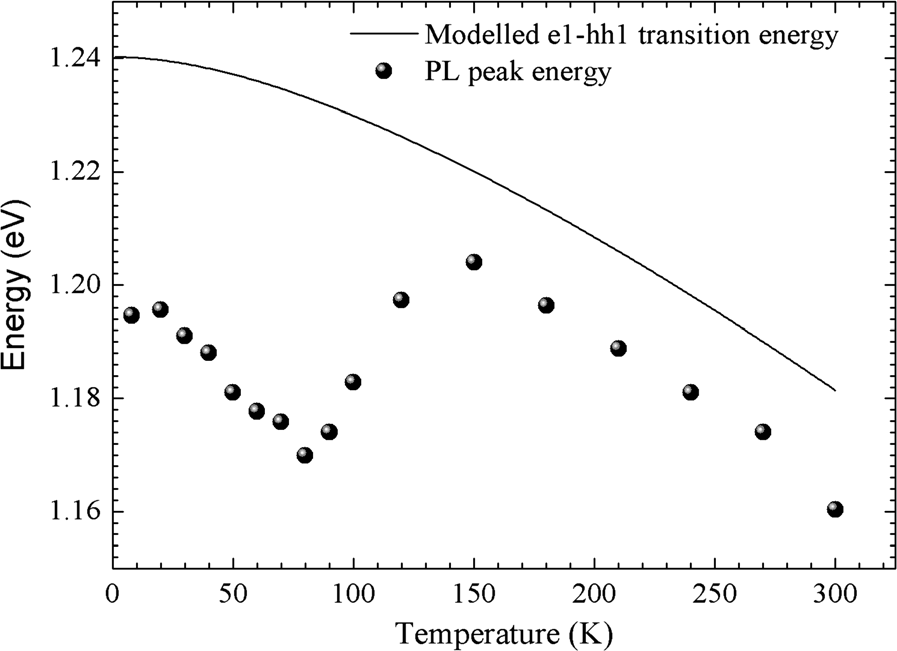
**The s**-**shape PL shift of the investigated sample.**

Spectral photoluminescence (PL) measurements were performed by illuminating the sample with the *λ* = 647 nm of a krypton ion laser and collecting the resulting PL signal with a N_2_-cooled GaInAs photomultiplier. The PL spectrum taken at *T* = 100 K is shown in Figure 1 and shows a very broad GaInNAs-related peak at *λ* = 1.080 nm. The distinct tail extending from the low energy side of the QW PL emission indicates strong carrier localisation where electrons become trapped in states below the conduction band and recombine radiatively [16,17]. The evidence for the recombination via the localised states comes from the observation of the well-known s-shaped behaviour of the temperature dependence of the PL peak [18] as depicted in Figure 2. In Figure 2, closed circles represent the expreimental results, and the continuous line is the theroretical fit for band-to-band recombination as explained in [18]. The experimental results have the same temperature behaviour as the theoretical calculations at *T* > 150 K. The approximately 10 meV difference, at these temperatures, between the experimental results and the theory may be due to the slight difference between the nominal value of nitrogen/indium concentrations as used in the theoretical calculations and the actual growth values. At temperatures *T* < 150 K, however, there is the well-known s-shaped behaviour associated with the radiative recombination via the localised nitrogen states below the conduction band.

**Figure 3:**
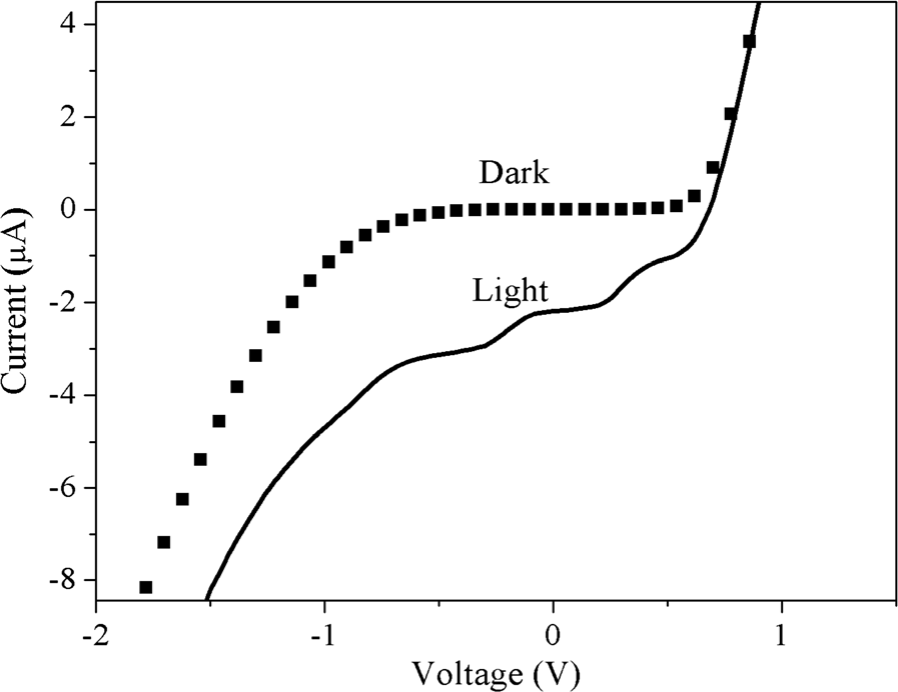
***I***-***V *results for the samples in dark and light conditions.**

The sample was characterised by measuring the *I*-*V* characteristics at *T* = 100 K in darkness and under illumination with *λ* = 950 nm LED. A Keithley 236 source measure unit (Keithley Instruments Inc., Cleveland, OH, USA) was employed in the experiments. In darkness, no oscillations were observed. However, after illumination, several oscillations appear in the *I*-*V* curve, both in forward and reverse bias as shown in Figure 3.

**Figure 4:**
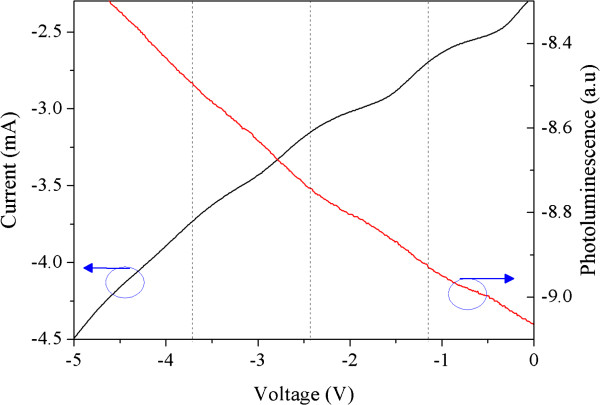
**PC and PLb for p**-**i**-**n GaInNAs MQWs at *T*** = **100 K.**

The integrated PL was recorded simultaneously with the PC signal by changing the applied reverse bias and using a specifically written acquisition programme. Because the optical excitation was provided with the krypton laser, a 900-nm high-pass filter was placed in front of the detector to remove the stray exciting light and the GaAs contribution. The integrated PL and PC results are plotted in Figure 4 as a function of the applied reverse bias. It is clear that the current oscillations also prevail in the PL signal, meaning that there is a strong link between photo-generated and emitted carriers.

**Figure 5:**
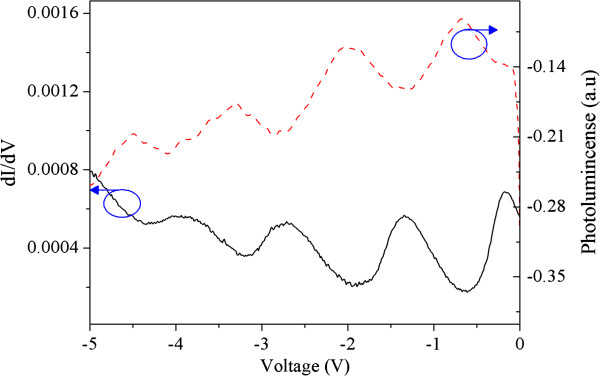
**The derivative of the photocurrent for PC and PLb experiment at *T*** = **100 K.**

The oscillations are much clearly visible in Figure 5, where the first derivative of the photocurrent and the PLb signals are plotted against the bias voltage. It is clear that a peak in the PC signal corresponds to a trough in the PLb, and vice versa. Therefore, each peak in the *IV* characteristics corresponds to the loss of carriers that would otherwise contribute to the radiative recombination. Therefore, the increase in photocurrent is accompanied with a decrease in the PL intensity. At the lattice temperature of around *T* ≈ 100 K, the current oscillations in the *IV* curves have their maximum amplitude, and they disappear completely at *T* > 220 K [15,19]. We have performed this experiment at different temperatures from 2 to 300 K. However, we focus our attention to the *T* = 100 K because at this specific temperature the oscillations present their maximum amplitude.

Under illumination, accumulation of negative photo-generated electrons will occur in the biased device because of the disparity between the hole thermal escape times from the quantum wells. The band offset (for conduction or valence bands) determines strongly the thermal escape times (*τ*_th_) described as [20]:

(1) $$\frac{1}{\tau_{\mathrm{th}}}=\frac{1}{L}\sqrt{\frac{K_{B}T}{2\pi m_{w}^{*}}}\exp\left(-\frac{E_{\mathrm{barrier}}}{k_{B}T}\right)$$

where $m_{w}^{*}$ is the effective mass for the carriers in the well; *E*_barrier_ is the energy difference between the sub-band and the barrier; and *L* is the well width.

**Figure 6:**
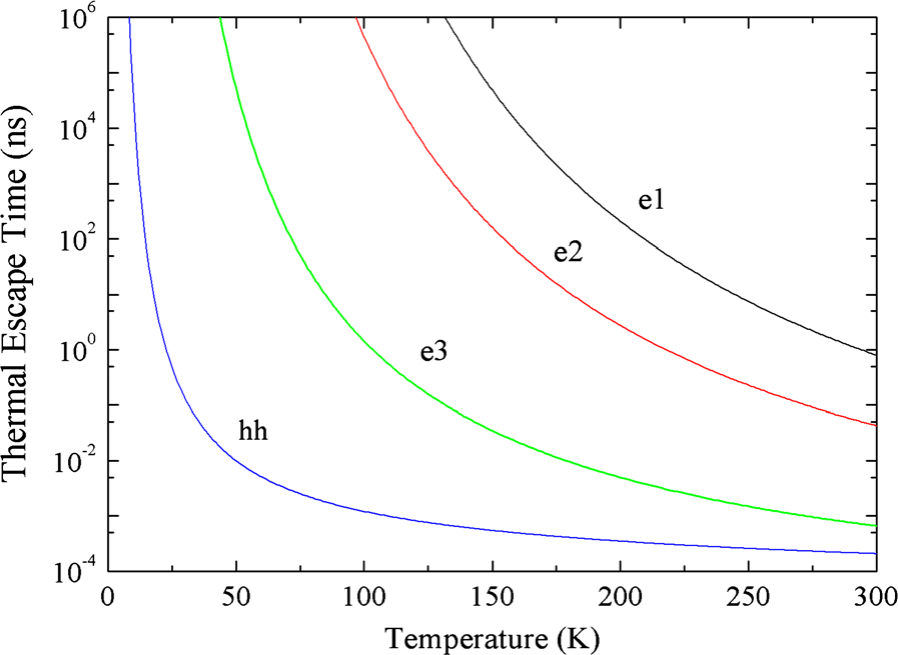
**Temperature dependence of the thermal escape times of electrons and heavy holes in the QW.**

Thermal escape times of electrons from sub-bands, *e*_1_, *e*_2_ and *e*_3_, and heavy holes in the quantum well are calculated using Equation 1 and plotted as a function of temperature in Figure 6. It is clear that the escape time for heavy holes is very short, around 2 × 10^−13^ s at room temperature as a result of the low valence band offset (*E*_barrier_ in Equation 1) [21]. The thermal escape time of heavy holes is 2 orders of magnitude shorter than the thermal escape times of *τ*_th_ = 6 × 10^−11^ s for electrons in the *e*_1_ band and an order of magnitude faster than *τ*_th_ = 4 × 10^−12^ s for electrons in the *e*_2_ band. As the temperature is decreased, the thermal escape time of electrons rapidly increases, while for holes, the time is less than 1 ns up to a temperature of ≈30 K. This is the result of the small valence band offset and thus the enhanced thermal escape rate for holes compared to the conduction band electrons. Therefore, photo-generated holes are rapidly thermally excited from the well and swept away under the influence of high electric fields. This results in the accumulation of negative charge in the QWs and breakdown of the steady state condition. Charge accumulation (domain) results in across one well at a time, and each of the steps observed in the PC signal will be due to consecutive carriers escaping from the well [22].

**Figure 7:**
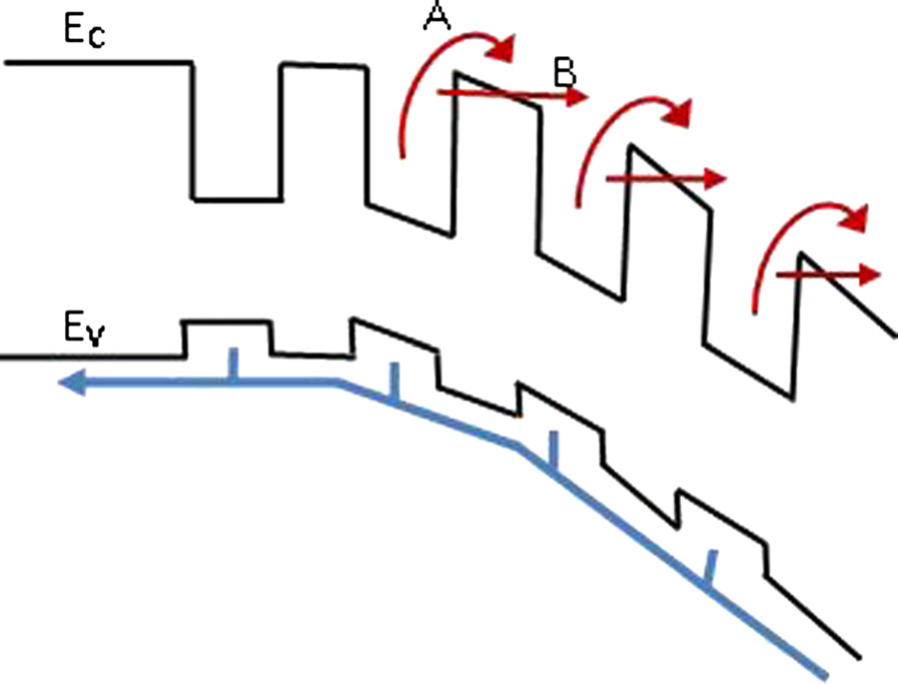
**Schematic illustration of the transport in illuminated and biased MQW p**-**i**-**n structure.** The blue arrows denote a negative charge, while the red arrows denote a positive charge.

In summary, the origin of photocurrent and PLb oscillations can be explained in terms of thermionic emission and Fowler-Nordheim tunnelling [23] through the triangular barrier due to the negative charge accumulation in the QWs. This process is illustrated in Figure 7 as A and B, respectively.

## Conclusion

Photocurrent and integrated photoluminescence measurements on a GaInNAs/GaAs multi-quantum well based p-i-n diode are performed at *T* = 100 K as a function of applied bias. The analysis reveals that under reverse bias, clear oscillations in the PC and PLb signals are observed. The difference in the thermal escape time of electrons and holes causes the accumulation of negative charge in the wells giving rise to the observed current oscillations.

## Competing interests

The authors declare that they have no competing interests.

## Authors’ contributions

HMK carried out the experimental work in collaboration with BR and SM. NB is the supervisor of the project. All authors read and approved the final manuscript.

## Supplementary Material

Additional file 1 The design of the p-i-n MQW sample. Click here for file
