# Supplementary material for: Photoconductivity and photoluminescence under bias in GaInNAs/GaAs MQW p-i-n structures
Source: Nanoscale Res Lett. 2012 Sep 28;7(1):539. doi: 10.1186/1556-276X-7-539 (PMC3479062; doi:10.1186/1556-276X-7-539)
Supplement: Additional file 1 — The design of the p-i-n MQW sample. [file 1556-276X-7-539-S1.pdf]

Dear editorial board of Nanoscale Research Letters,
Please find enclosed the manuscript:"Photoconductivity and photoluminescence under bias in GaInNAs/GaAs MQW p-i-n structures", by Hagir M. Khalil, et al., to be submitted as an article after poster presentation at the International Conference on Superlattices, Nanostructures, and Nanodevices (ICSNN 2012) , Dresden, Germany. All co-authors have seen and agree with the contents of the manuscript and there is no financial interest to report. We certify that the submission is original work and is not under review at any other publication.
We hope that the editorial board will agree on the interest of this study.

Sincerely yours,
H. Khalil on behalf of the authors.
Corresponding author: Hagir Mohammed Khalil,
School of CSEE,
University of Essex
Colchester, Essex
CO4 3SQ
UK
hkhalia@essex.ac.uk,
Tel: +44 1206 87 2286.
